# Influence of Natural Risks and Non-Agricultural Income on Agricultural Trusteeship Decisions in Northeast China

**DOI:** 10.3390/foods13132024

**Published:** 2024-06-26

**Authors:** Ying Xue, Hongbin Liu

**Affiliations:** 1 College of Land and Environment, Shenyang Agricultural University, Shenyang 110866, China; 2 College of Economics and Management, Shenyang Agricultural University, Shenyang 110866, China

**Keywords:** natural risks, NAEI, APTS, farmers, food security

## Abstract

As the main service mode and management mode of socialized service, agricultural production trusteeship services (APTS) are an important way to realize the tight link between farmers and modern agricultural development, which is highly important for ensuring national food security. By constructing a utility model of farmers, this paper deduces the decision-making mechanism of farmers’ APTS from the dual mechanism of natural risk and non-agricultural employment income (NAEI) and uses the survey data of 956 farmers in the three northeastern provinces to verify the empirical results by using multivariate logit (Mlogit) and propensity score matching (PSM) methods. The research shows that natural risk hinders farmers’ choice of the APTS; NAEI has a positive impact on APST, and an increase in NAEI weakens the negative effect of natural risk on the choice of the APTS, and has an enhanced moderating effect. In addition, the scale heterogeneity analysis revealed that the impacts of natural risk and NAEI on farmers of different scales are significantly different. The NAEI adjustment effect has the greatest impact on small-scale farmers, followed by medium-scale farmers. In view of this, the government should stabilize the nonagricultural employment market and improve the nonagricultural employment opportunities of farmers. APTS organizations should transfer natural risks and improve the agricultural insurance system. According to the differences of different scales of farmers, different promotion policies are formulated, and the expansion of management and deepening services is proposed to further promote the development of APTS and consolidate the foundation of food security in China.

## 1. Introduction

In the face of the reality of the low level of agricultural scale operation [1], high cost of grain production [2,3], and lack of a young and middle-aged labor force in rural areas [4,5,6], how to realize the transformation of agricultural operation modes and the improvement of grain production efficiency has become an important problem to be solved urgently [7,8,9]. The development of APTS and the promotion of service scale operation are the keys to answering this question [10]. As an important part of agricultural socialized service or agricultural productive service, APTS is an agricultural management mode in which farmers and other business entities independently entrust all or part of the farming, planting, prevention, and harvesting in agricultural production to service organizations or individuals to complete under the condition of retaining land management rights, mainly for the production link [11,12,13]. As an innovation of the management mode, by involving small farmers in the socialized division of labor, it can realize the scale management of services, promote the tight link between small farmers and modern agricultural development, and play an indispensable role in food and agricultural production [14]. With the support and guidance of a series of relevant policies such as “the Guiding Opinions on Accelerating the Development of Agricultural Producer Services” enacted in 2017 by The Ministry of Agriculture, the National Development and Reform Commission and the Ministry of Finance, China’s APTS has developed rapidly, and the economic effects of service scale operation have gradually emerged [15]. Different from the path of promoting large-scale land management through land transfer, APTS can supplement and answer the questions of “who will farm”, and “how to farm”, effectively alleviating the problems of “being unable to farm” and “bad to farm” under the premise of maintaining the distribution of land management rights, and promoting the development of service-driven large-scale operation, thus solving the current inherent contradiction between “small production” and “big market” [16]. According to the characteristics of China’s agricultural development, the APTS has overcome the bottleneck of small farmer grain planting, promoted the unity of grain production and quality improvement, and gradually become the main way to stabilize the strategic foundation of China’s food security.

However, the rapid development of APTS in China is also accompanied by risk problems. Among them, natural disasters have become a major constraint that hinders farmers from choosing different agricultural production services [17,18]. Natural risk is an important problem often faced in China’s agricultural production, and it is the most important risk factor affecting agricultural production [19]. Research on natural risks mainly focuses on three aspects: first, to explore the methods of assessing agricultural risks [20,21]; second, it is to discuss the impact of climate change on agricultural production output at the macro level [22,23]; third, it is to analyze the impact of relative poverty brought about by natural risks from the perspective of rural households, impact on technology adoption [24], etc. It can be found that few studies have explored the relationship between natural risks and farmers’ agricultural production trusteeship decisions [25]. In the survey, it was found that when natural risks occur, farmers will experience disputes with trustees because of production. At this time, farmers tend to rely on their own strengths to avoid climate disasters and rely less on external forces. How to avoid the impact of natural risks on the decision-making of the APTS is the focus of this paper. An effective way to improve farmers’ ability to resist natural risks is to increase farmers’ income [26]. With the rapid development of the economy, China’s nonagricultural sector has grown rapidly, and the outflow of labor has become a trend, which has greatly increased the employment opportunities of farmers and become an effective means for farmers to increase their income [27]. The deepening of farmers’ degrees from agricultural production to non-agricultural concurrent industry is a manifestation of the deepening division of labor. To a certain extent, it can overcome the disadvantages brought about by the small-scale peasant economy in which traditional families make their own decisions. Compared with ordinary farmers, non-agricultural employment farmers have relatively strong anti-risk abilities in agricultural production. Therefore, the NAEI may have the ability to solve the problem of the APTS selection constraints caused by natural disasters.

Reviewing the literature, on the issue of exploring the factors affecting APTS decision-making, scholars have carried out in-depth research on different aspects, such as individual characteristics [28,29], family characteristics [30], land trusteeship prices [31], the number of agricultural machineries [32], agricultural operations [33], and land trusteeship cognition [16]. Some scholars have focused on the impact of natural risks on farmers’ APST decision-making [34]. However, due to the limitations of the research perspective and sample data, no consensus has been reached on its effect. Some studies suggest that farmers’ risk aversion hinders the spread and adoption of agricultural innovation, although these innovations can increase the yield and income of farmers, resulting in inefficient use of resources at the farmer level [35,36,37]. In the face of natural disasters, its resilience is weaker than that of other groups. However, some studies believe that natural risk perception has a positive effect on farmers’ participation in APTS [38]. Natural risk perception affects APTS decision-making through perceptual behavior control. Subjective norms can enhance the induction effect of farmers’ risk perception, which in turn can promote farmers’ choice of APTS [38,39]. In summary, the existing literature provides experience for further research in this paper, but there are still some shortcomings, which are manifested in the following aspects: First, from the perspective of research, natural risk, is the most difficult factor for farmers to control in agricultural production, is a factor that agricultural producers focus on when conducting agricultural production. At present, the research on natural risks is more from a macro perspective, and there are relatively few studies on agricultural production decision-making behavior from the micro perspective of farmers, and the conclusions are not yet unified. Secondly, in terms of research content, existing research has focused on the impact of natural risks and non-agricultural employment on the APTS, but analyses of the impact path and impact mechanism are relatively lacking. There is a lack of research on subdividing different types of APTS, and the adjustment effect of the NAEI on natural risks is rarely concerned. Third, in terms of research methods, some studies ignore the possible sample selection bias and endogeneity problems, resulting in a certain impact on the accuracy of the estimation results.

In view of this, based on the theoretical model of farmers’ utility, this study constructs a theoretical analysis framework for the decision-making of APTS, explores its influence mechanism on farmers’ APTS choice behavior from the perspectives of natural risk and the NAEI, and clarifies the moderating effect of NAEI on natural risk. Using the disordered Mlogit and PSM econometric model and survey data from 956 corn growers in the typical region of the black soil region in Northeast China, this paper empirically tests the internal mechanism of the impact of natural risks and the NAEI on the decision-making of the APTS. The focus of this study is to answer the following questions: First, how does natural risk affect farmers’ decision-making regarding APTS? Second, can the NAEI effectively regulate the impact of natural risks on the choice of APTS? The research results can further enrich the theory of APTS selection behavior of micro farmers, help to expand the vision and scope of scientific research, and deepen the existing research contents and methods. The policy significance is to provide theoretical and empirical evidence for further accelerating the development of APTS in the black soil area of Northeast China and formulating diverse, flexible, and effective incentive policies. This study provides theoretical reference and practical basis for reducing the risk of natural disasters, realize the tight link between farmers and modern agricultural development, and consolidating the win-win basis of food security.

## 2. Theoretical Framework

### 2.1. The Influence Mechanism of Natural Risk on Farmers’ APTS Adoption

According to the theory of new family economics, farmer production is ultimately used to consume goods and services, and the family utility function represents the family’s preference for this series of goods and labor [40,41,42]. With respect to APTS, the ultimate goal of farmers’ production decisions is to maximize family utility. Therefore, based on the theory of the Barrum-Squier farmer model [43,44], this paper modifies the farmer model and establishes a decision-making model for farmers to choose the APTS. The basic principle of constructing a new farmer model is to maximize family utility under the three conditions of family production function, total labor time constraint, and investment capital constraint. 

It is assumed that there is no non-agricultural employment market, and that all the agricultural products produced by farmers are sufficient for household consumption. Corn production consists of five steps: soil preparation, sowing, fertilization, field management, and harvesting. The employment service cost (including transaction cost) of each link per unit of time is *W*, and the number of custody links is *n*, *n* = 1, 2, …… 5. Constructing a farmer utility model, the specific form is as follows:

(1) $$U=f(Y,nWT_{2},T_{S})$$

(2) $$s.t.\;Y(TPP_{1})=f(A,L,K,nWT_{2})$$

(3) $$T\leq T_{S}+T_{1}+T_{2}$$

(4) $$p(H-Q_{0})\leq N+nWT_{2}$$

(1) is the family utility function, where *Y* represents the production function of farmers; *T_s_* is the labor time of farmers, and *nWT*_2_ is the total cost of farmers’ trusteeship. Formula (2) is the production function constraint of farmers [45], in which the corn planting area of farmers is *A*, the agricultural machinery endowment of farmers is *K*, and the labor endowment of farmers (excluding leisure time) is *L*. Equation (3) represents the time constraint, where *T* represents the total time of family labor, *T*_1_ is the time of family agricultural production, and *T*_2_ represents the time of hired labor. (4) The type of financial constraint, refers to the family’s production income being equal to the means of production expenditure and custody service expenditure. *P* is the price of agricultural products, Q is the output of agricultural products, and $Q_{0}$ is the output of farmers for self-consumption. $\left(Q-Q_{0}\right)$ is the output of agricultural products sold. 

**Figure 1:**
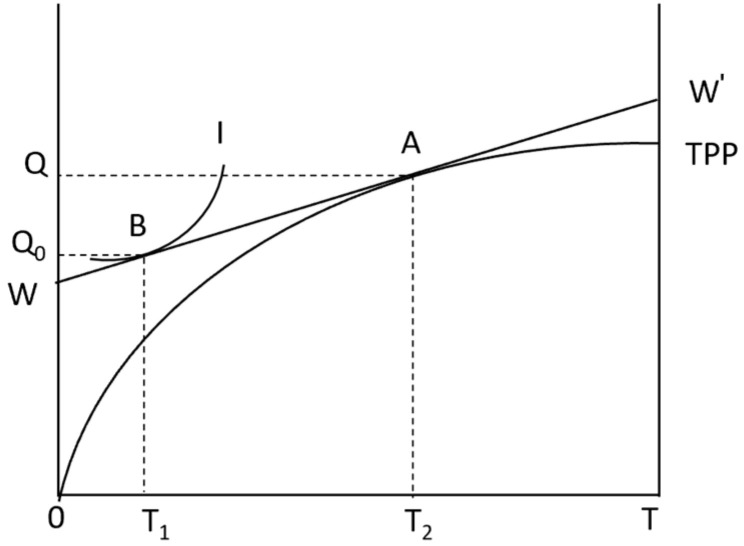
Farmer utility model.

According to the above utility function and its constraint conditions, the following analysis is carried out:

Without considering natural risk, the utility model of farmers is constructed as shown in Figure 1. The horizontal axis represents the total time *T* that farmers can control, and the vertical axis represents the corn yield Q. *TPP*_1_ is the production function of farmers’ choice of managed services to produce corn. *I* represent the indifference curve, which represents the specific utility level brought by the different combinations of farmers’ leisure time and income to the family. In other words, the indifference curve reflects the farmers’ value of time. Willingness and the slope of any point on the indifference curve are the marginal substitution rates of income and leisure. $WW^{\prime }$ represents the wage cost of farmers in agricultural production, that is, the wage rate of employment services. As shown in Figure 1, the equilibrium point of agricultural production is *A*, and the equilibrium point of consumption is *B*. At this time, the distance $\left(T_{2}-T_{1}\right)$ between *T*_1_ and *T*_2_ is the time when farmers choose hosting services.

**Figure 2:**
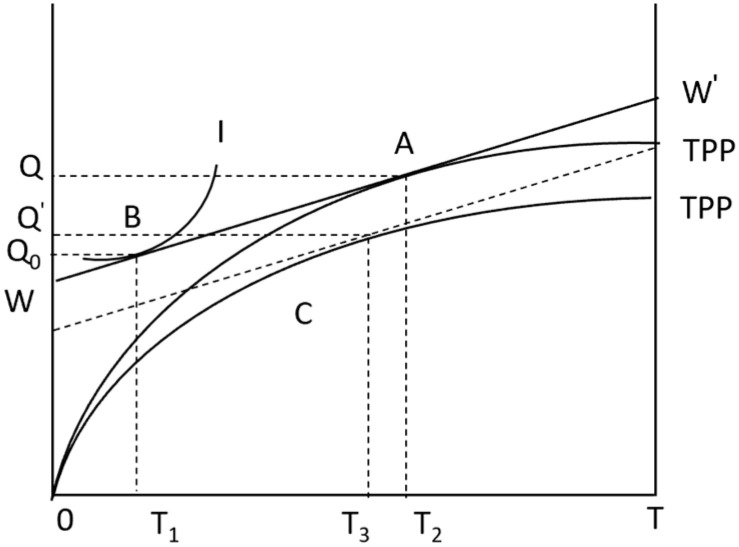
Farmer utility model under natural risk.

Considering the factors of natural risk, the farmer’s utility model is constructed as shown in Figure 2. In agricultural production, the uncertainty of agricultural production and price caused by weather, market, and technical factors will eventually manifest as fluctuations in agricultural production income. Under the same income level, the greater the fluctuation of income obtained by farmers engaged in agricultural production, the greater the production risk faced by farmers; correspondingly, in the case of a certain range of income fluctuations, the higher the income of farmers engaged in agricultural production activities, the lower the risk of agricultural production faced by farmers [46,47,48]. When there is natural risk, it is assumed that the probability of risk under natural risk conditions is *P_r_*($0\leq P_{r}\leq 1$), and the natural risk faced by farmers is short-term, which has little impact on farmers’ production utility. At this time, the production function of farmers is: $Y(TPP_{2})=f(A,L,K,nMT_{2},P_{r})$.

The production income of farmers will fluctuate with the risk probability, and the production function curve will decline from the original *TPP* to *TPP*_2_ (Figure 2). At the same time, a new production equilibrium point *C* is formed by $WW^{\prime }$. $T_{3}-T_{1}$ is the trusteeship service time that farmers need to purchase under the influence of natural risks. $T_{3}-T_{1}<T_{2}-T_{1}$ shows that under the influence of natural risks, farmers will reduce their choice of APTS. In other words, natural risks inhibit farmers’ choice of APTS adoption. Therefore, research Hypothesis 1 is proposed:

**H1.**  *In the face of natural risks, farmers will inhibit their choice of the APTS.*

### 2.2. The Influence Mechanism of the NAEI on Farmers’ APTS Adoption

Regardless of the risk, when there is a non-agricultural employment market, it is assumed that the wage rate of the non-agricultural employment market is the same as that of the employment service. All are $WW^{\prime }$. At this time, in the original assumptions, farmers’ self-sufficiency in production has changed. Farmers need to sell some products to purchase non-agricultural consumer goods, that is, industrial goods *M*. An important choice for farmers is how many products are used for self-consumption $\left(Q_{0}\right)$ and how many products are used for sale. 

**Figure 3:**
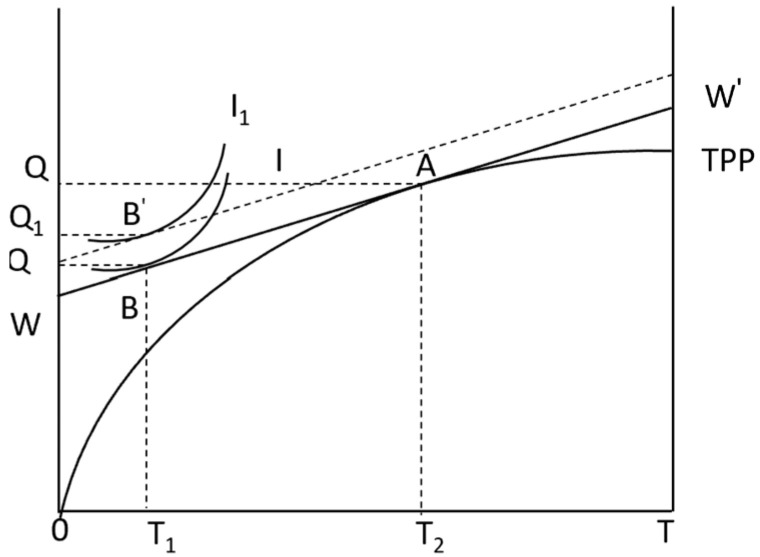
Strongly hypothetical the NAEI utility mode.

The indifference curve represents the substitution rate of income and leisure. If there is a non-agricultural employment market, the change in the indifference curve will inevitably change. To facilitate the analysis, it is strongly assumed that the increase in the NAEI does not change the shape of the indifference curve $I_{1}$ (Figure 3). The relationship $I_{1}$ with I: do not change the shape, parallel up. At this time, the output did not change, that is, *Q* did not change, but the output of agricultural products retained by farmers increased $\left(Q_{1}-Q_{0}\right)$. At this time $\left(Q-Q_{1})<(Q-Q_{0}\right)$, the constraint condition of Equation (4) is not satisfied, that is, the output income sold cannot buy employment services, so farmers save time entering the non-agricultural employment market and earn non-agricultural wages $W_{Z}$ to obtain more income. 

Farmers’ utility model in the non-agricultural employment market has the following changes:

(5) $$U_{1}=f(Y,T_{z}W_{z},nWT_{2},mM,T_{S})$$

(6) $$s.t.\;T\leq T_{S}+T_{1}+T_{2}+T_{z}$$

(7) $$p(Q-Q_{2})+T_{Z}W_{Z}>=N+nWT_{2}+mM$$

**Figure 4:**
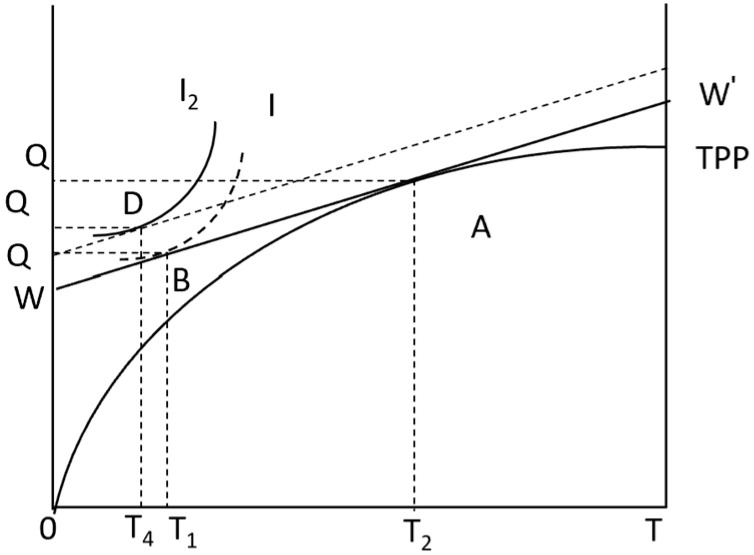
Farmers’ utility model under the NAEI.

The family utility function of Equation (5) increases $T_{Z}W_{Z}$ and mM, where $T_{z}$ represents the time of migrant employment of farmers; $W_{Z}$ represents the average wage of farmers’ out-migration employment, and $T_{Z}W_{Z}$ represents the cost of production materials invested in the production of the opportunity cost of farmers’ non-agricultural employment. *m* is the average price of commodity *M* in in the market. When (6) joins the non-agricultural employment market, the time constraint changes. According to Equation (7) income constraint refers to the net income of the family being equal to the total expenditure of the family for the purchase of market goods. Under this constraint condition, when farmers have non-agricultural employment, the indifference curve rotates to the right to form a new one $I_{2}$ (Figure 4). A new equilibrium point *D* is formed by $I_{2}$ and $WW^{\prime }$, where $Q_{2}$ represents the agricultural products consumed by farmers themselves under the new constraints.

Figure 4 shows that if farmers want to increase the NAEI, they can only carry out non-agricultural work by reducing agricultural production labor. Under the above constraints, the agricultural production time of farmers decreases from T_1_ to T_4_, and the time of employment service increases, that is, $\left(T_{2}-T_{4})>(T_{2}-T_{1}\right)$. It can be inferred that an increase in the NAEI will encourage farmers to choose APTS. Based on the above analysis, this paper proposes Hypothesis 2:

**H2.**  *The NAEI has a positive impact on the choice of APTS.*

### 2.3. The Moderating Effect of the NAEI on the Influence of Natural Risk on Farmers’ APTS Choice Behavior

**Figure 5:**
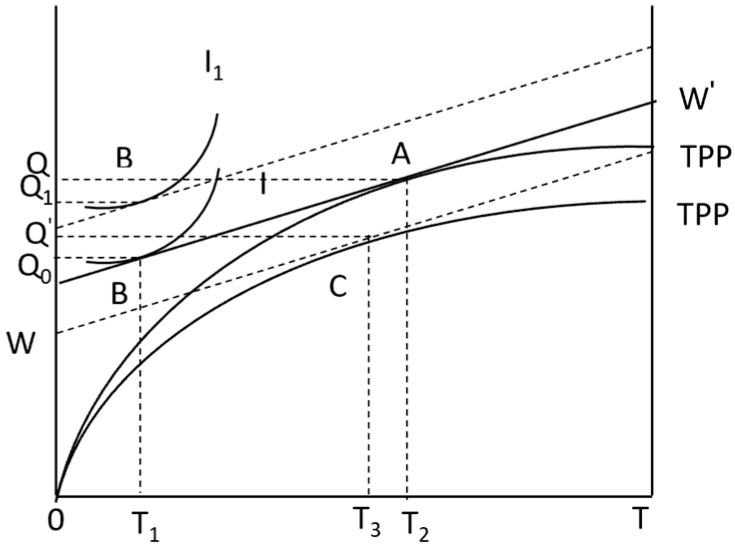
The utility model of farmers’ participation in the NAEI under the assumption of natural risk.

**Figure 6:**
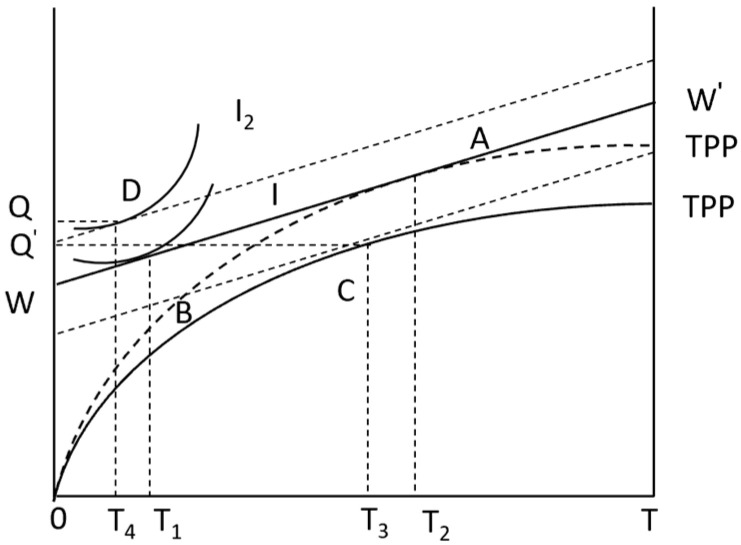
The utility model of farmers’ participation in the NAEI under natural risk.

On the basis of the above analysis, when there is a non-agricultural employment market, and taking into account natural risk factors, a farmer utility model is constructed as shown in Figure 6. Compared with Figure 2, when natural risks occur, the wage rate in the non-agricultural employment market is not affected. Therefore, *TPP*_2_ remains unchanged and the indifference curve moves. Firstly, on the basis of Figure 2, it is strongly assumed that the indifference curve does not change the shape, but I  moves up from parallel to $I_{1}$ (Figure 5). At this time, the equilibrium $P(Q^{\prime }-Q_{0})=N+nWT_{3}$ of the income constraint line under the natural risk of Figure 2 is broken, forming a new equilibrium point $B^{\prime }$. The agricultural products produced $\left(Q^{\prime }\right)$ by farmers cannot meet the needs of farmers’ families $\left(Q_{1}\right)$, and even need to purchase agricultural products $\left(Q_{1}-Q^{\prime }\right)$. 

When farmers join the non-agricultural employment market and obtain the NAEI, the indifference curve will rotate to the right, forming a new equilibrium point *D*, and the demand for agricultural products consumed by farmers themselves decreases to $Q_{2}$. At this time, the income constraint is $p(H-Q_{2})+T_{Z}W_{Z}>=N+nWT_{2}+mM$:. Farmers will increase their non-agricultural employment $\left(T_{1}-T_{4}\right)$, increase their income, and meet their family life needs. Figure 6 shows that in the face of natural risks, although farmers want to reduce costs by reducing employment services, under the intervention of non-agricultural markets, farmers may increase the NAEI to meet the same utility. This shows that the NAEI of farmers can weaken the negative impact of natural risks on APTS adoption by increasing farmer incomes. Based on the above analysis, this paper proposes research Hypothesis 3.

**H3.**  *The NAEI plays a positive moderating role in the influence of natural risk on farmers’ APTS choice behavior.*

## 3. Materials and Methods

### 3.1. Data Source

The data of this study are derived from the survey of corn growers in the three northeastern provinces by the research group from July to August 2018. Although these data are from 2018, they are still valid and valuable because research results on similar topics have been published [49,50,51,52]. The reasons for using the northeast region as the research area are as follows: First, as one of the three black soil regions in the world, the northeast region is an important commodity grain base in China. The grain output accounts for 1/4 of the country, the commodity volume accounts for 1/4 of the country, and the export volume accounts for 1/3 of the country. It is a ballast stone and regulator of national food security. According to the official statistics of the China Bureau of Statistics, in 2022, the total sown area of crops reached 25,762,600 hm^2^, accounting for 15.2%, of which the corn sown area accounted for 30.6%. Second, the level of mechanization in Northeast China is developing rapidly. The total power of agricultural machinery in Northeast China will reach 1.4 × 10^8^ W, accounting for 12.75% of the total power of agricultural machinery in China. At the same time, with the development of mechanization, social service organizations in Northeast China are gradually growing, and diversified business entities are providing l productive agricultural services; however, this development is not standardized, and it needs to be guided to ensure it healthy and long-term development. Third, the disaster-free area in Northeast China accounted for 14.5%, and the affected area accounted for 9%. Northeast China, as one of China’s important grain production bases, is affected by natural disasters. Based on the above analysis, it is typical and representative to choose the northeast region as the research area.

Considering that the development level of agricultural production hosting services is very different, a total of 6 cities were selected in Liaoning, Jilin and Heilongjiang provinces by typical sampling method. Among them, Harbin, Qiqihar and Suihua were selected in Heilongjiang Province; Changchun City and Siping City were selected in Jilin Province; Tieling City was selected in Liaoning Province. In each sample city, 2~3 townships were selected by random sampling, and a total of 16 sample townships and 43 villages were selected. The survey takes a one-to-one form to fill in the questionnaire, and the survey subjects are farmers. Most of the respondents were heads of farmers. When the heads of farmers were not at home, family members who had a greater impact on family decision-making were selected as respondents. The survey included the basic information about the family population, family production and operation, agricultural production links, APTS selection, etc. and956 valid samples were ultimately obtained.

### 3.2. Methods

The disordered Mlogit model is another natural continuation of a binary logit model [53]. The independent variables of the model can be continuous variables [54,55,56,57], discrete variables, or dummy variables, and a multivariate normal distribution between the independent variables is not needed. It can address the case where the explained variable is a categorical variable. The advantage of this approach is that the calculation is simple and the hypothesis test is convenient; additionally, this approach has advantages in obtaining the difference between each group category and the base group. When the dependent variable is a dummy variable or a numerical variable, the estimation results are still consistent and effective, and the ordinary least squares estimation results are biased. In this study, the choice behavior of farmers’ APTS is taken as the dependent variable of the study, including three kinds of choice behaviors: self-cultivation, partial link APTS and whole-process APTS, and there is no ordered relationship between them. These three selection behaviors are multi-categorical and have no sequential relationship with each other. This is very consistent with the requirements of the Multinomial logistic model. Therefore, according to the research content of this paper, the construction model is as follows:

(8) $$P(y_{i}=j|x_{i})=\frac{\exp(x_{i}^{\prime }\beta_{j})}{\sum_{k=1}^{J}\exp(x_{i}^{\prime }\beta_{k})}$$

P in Equation (8) represents the probability that the i farmer will choose the j APTS. $x_{i}$ expressed the explanatory variables such as natural risk and the NAEI. β represents the coefficient to be estimated, and k represents the number of agricultural productive services available for farmers to choose. The disordered multiple regression selection model can not identify all the coefficients $\beta_{k}$ to be estimated at the same time, so $y_{i}=k$ assumes that the control group is compared and estimated, and Equation (8) is sorted out and deformed, and the final behavioral model of APTS is as follows:

(9) $$\ln(\frac{P_{j}}{P_{J}})=\beta_{0}+\beta_{1}Disaster+\beta_{2}Nonfarm+\beta_{3}Disaster*Nonfarm+\sum \beta_{i}Z_{i}+\mu$$

In Equation (9), *Disaster* represents’ whether the year is affected by natural disasters’ to measure natural risks. *Nonfarm* represents’ the average annual NAEI of the family to measure the NAEI, and *Disaster* ∗ *Nonfarm* represents the interaction term between natural risks and the NAEI. Z is a control variable, and μ is a random error term.

### 3.3. Variable Selection

#### 3.3.1. Explained Variables

Whether farmers choose APTS is the dependent variable in this paper. The APTS can be divided into two types according to the different service links. One is the partial link APTS, also known as the partial link APTS; the other is the whole-process APTS. The market capacity for agricultural production hosting services is sufficient. Every farmer has the right to choose between agricultural production custody and non-agricultural production custody services. Farmers first consider whether to choose APTS, and then they will consider which type of service to choose. Therefore, the explanatory variables assigned to the model in this paper are as follows: self-cultivation without APTS, with a value of 0; select the part partial link APTS, assigned to 1; and whole-process APTS, assigned to 2.

#### 3.3.2. Key Variables

The core explanatory variables of this model are the proxy variable of natural risk and the NAEI variable. The index of natural risk is usually macro data in the general literature, which is calculated by the disaster situation. At this time, the natural risk represents the risk of a certain area, and the risk faced by each farmer is the same. However, the natural risk in this paper refers to natural disasters such as drought, wind and floods that family production may encounter, which are measured at the micro level. Through investigation, it was found that there are many kinds of natural risks in corn production in Northeast China, even within the same plot, the situation is not the same. Therefore, in APTS, the risks faced by farmers, which are individual variables are different. This paper uses whether natural disasters occurred last year as an indicator of natural risk. There are two points: First, Frank Ellis (1993) believes that risk can be measured by probability [43]. Under the assumption of rational expectations, farmers can rationally expect natural risks. However, it is difficult to reveal the impact of natural risks on farmers’ APTS selection behavior under the current technical methods. In previous studies, scholars used the number of natural disasters in recent years as a proxy variable for natural risks [58,59,60], and there is a deviation in farmers’ understanding, which is considered to be the number of natural disasters in the region. The choice of the variable whether there was a natural disaster in the previous year can directly indicate the impact on service selection in that year. Therefore, it is more reasonable to use whether natural disasters occurred last year to examine the choice of natural risks to APTS. It is necessary to consider the division of labor within the family when examining the NAEI by farmer units, so it is more reasonable to replace the NAEI with the per capita non-agricultural annual income of the family [61].

#### 3.3.3. Control Variables

Based on the relevant practices of the literature [36,62,63,64], this paper mainly chooses the individual characteristics of the head of farmer and the characteristics of farmers as the control variables (Table 1). In the process of agricultural production, the head of farmers, as the main body of production decision-making and the main labor force of the family, usually has high family authority. Difference in individual characteristics may affect the decision-making of agricultural production and the purchase of managed services. Age, years of education, risk preference, etc., are selected as alternative variables for the individual characteristics of the head of farmers. As an embedded resource, family resource endowment can affect farmers’ agricultural production decision-making, and subsequently affect agricultural output. The family net income, family production capacity, agricultural machinery and relationship network are selected as the substitution variables of family characteristics. In addition, considering the differences between regions, this paper also introduces regional and provincial dummy variables.

## 4. Results

This paper uses the field survey data of one-to-one farmers in the three northeastern provinces to conduct multiple Mlogit regression analyses to analyze the impact of natural risks on farmers’ APTS choices and the adjustment effect of the NAEI on the impact of natural risks on farmers’ APTS choices. To avoid multicollinearity, the moderating effect centralizes natural risks, the NAEI and control variables before testing the interaction effect. Table 2 and Table 3 show the estimation results of the impact of natural risk on farmers’ APTS choices and the estimation results of the adjustment effect of the NAEI. To control for the possible influence of heteroscedasticity, autocorrelation and outliers in the model disturbance term, this paper adopts robust estimation for all regressions.

### 4.1. The Impact of Natural Risks on APTS Choice

To avoid the influence of multicollinearity, hierarchical regression is used. First, the influence of the natural risk substitution variable and the NAEI variable on APTS adoption is considered separately, and Model 1 and Model 2 are obtained. Second, the natural risk variables and the NAEI variables are included in the regression, and Model 3 is obtained. The regression results of models 1~3 are shown in Table 2.

Table 2 shows that natural risks have a significant impact on APTS, regardless of whether the NAEI variables are added, and the direction is negative. That is, the greater the expected natural risk of farmers is, the more inclined they are to cultivate themselves; that is, natural risk is an obstacle to farmers’ choice of APTS, which verifies Hypothesis 1.

Further analysis revealed that natural risks have a significant negative impact on the partial link APTS of farmers, Specifically, compared with farmers who are self-cultivation, the probability of natural risk for farmers to choice of partial outsourcing services decreases by 53.57% [65,66,67]. At the same time, natural risks have little effect on farmers’ choice of partial outsourcing services or whole-process hosting services, both of which have a hindrance effect. Compared with self-cultivation, the probability of natural risk for farmers to choose the whole process of custody service is reduced by 57.80%. Although the whole-process hosting service is branded with risk sharing, it is still unable to resist and no longer promises to ensure production if it encounters natural risks. Therefore, when farmers expect risks to occur, they will hinder the choice of APTS.

### 4.2. The Impact of Non-Agricultural Employment on APTS Adoption

From the estimation results of the explanatory variables of the NAEI in Model 2 and Model 3, the NAEI has a positive and significant impact on the choice of APTS; that is, the greater the NAEI of farmers is, the more inclined they are to choose APTS, and Hypothesis 2 is verified. The incidence of farmers with a high NAEI choosing the agricultural production part of the trusteeship service and the whole process of the trusteeship service is 52.40 times and 54.62 times that of farmers with low non-agricultural employment, respectively (Model 3). This shows that the level of the NAEI of farmers has no obvious effect on the choice of APTS type. The conclusions of previous studies are consistent [68]. The study also concluded that the percent of family members engaging in off-farm work has a positive effect on maize farmers’ agricultural mechanization services adoption. The results show that the increase of non-farm employment income will promote the development of agricultural production trusteeship services, which provides a basis for the government to formulate relevant policies.

According to the estimation results of control variables, among the characteristic variables of household heads, the age and education level of household heads have a significant positive impact on farmers’ choice of agricultural production custodian services, which is consistent with the relevant research results [69]. However, the degree of risk preference of the householder has a significant positive impact on the choice of the whole custody service, but has no impact on some link services. This is because the whole custody service is an emerging agricultural production service mode in recent years, and in the face of relatively high custody costs and the commitment to improve yield, some conservative farmers are still observing, and farmers’ risk appetite is an important constraint affecting farmers’ choice of full custody service. This is consistent with the conclusion of scholar Ahmad et al. [38]. For the variable of family resource endowment, whether a family has agricultural machinery has a significant negative impact on APTS. The stronger the family relationship network is, the greater the probability of choosing the APTS. The greater the fluctuation of farmers production output, the lower the agricultural production capacity of the family, and the more inclined farmers are to choose the whole-process APTS. In addition, the provincial variables and regional variables are significant, indicating that there are significant regional differences in APTS, especially whole-process APTS, and that the choices of these variables differ among different provinces.

### 4.3. The Moderating Effect of the NAEI on the Effect of Natural Risk on Farmers’ APTS Adoption

Some researchers have pointed out that the moderating effect is different from the interaction effect [70,71,72]. Among the moderating variables, which is the independent variable and which is the moderating variable is very clear. To test the moderating effect of the NAEI on the influence of natural risk on farmers’ APTS, and to reduce the multicollinearity of natural risk, the NAEI and the interaction term, this paper introduces the explanatory variable of natural risk and adds the interaction term of the NAEI and natural risk to the sample to obtain Model 5. Before the regression, considering that there may be a high correlation between the interaction term and the interaction term construction variable, the interaction term is constructed in a centralized direction, that is, the relevant variables are subtracted from the mean before the interaction term is constructed. The regression results are shown in Table 3.

From the estimation results of Model 5 in Table 3, the interaction term of natural risk and the NAEI has a positive impact on the choice of APTS. This shows that the NAEI plays a positive regulatory role in the negative impact of natural risk on farmers’ choice of APTS, that is, the NAEI weakens the negative impact of natural risk on farmers’ choice of APTS, which verifies Hypothesis 3. Specifically, compared with the self-cultivated farmers, non -agricultural employment income has increased by 1.89 and 1.81 times in the negative impact of natural risks for farmers to choice of partial outsourcing services and farmers to choose the whole process of custody services.

### 4.4. Robustness Test of the Model

Considering that farmers may have errors in self-selection, which may lead to unstable results, this paper uses the propensity score matching (PSM) method for matching estimation [73,74,75]. Table 4 shows the matching results of three different matching methods: the nearest neighbor matching method, the radius matching method and the kernel matching method. To facilitate the analysis, the continuous variables of the NAEI are converted into categorical variables, that is, 1 for those with the NAEI and 0 for those without the NAEI. According to the matching results in Table 4, the occurrence of natural risks may hinder the choice of APTS, and the acquisition of the NAEI has a positive impact on farmers’ choice of APTS, which is consistent with the previous hypothesis. Therefore, even if there is a certain self-selection bias problem, the impact on the measurement results will not cause too much interference, and the results are robust.

### 4.5. Discussion of Scale Heterogeneity

Considering that farmers of different sizes have different anti-risk capabilities, this paper attempts to divide the sample data according to different sizes and conduct regression analysis. According to the actual production of the sample area and the general definition of the literature [76,77,78,79,80], this paper defines the large-scale farmers as those with more than 50 acres, the medium-scale farmers as those with 20~50 acres, and small-scale farmers as those with 0~20 acres. Among the 956 sample farmers, 382 were small-scale farmers, 363 were medium-scale farmers, and 211 were large-scale farmers.

#### 4.5.1. The Impact of Natural Risks and the NAEI on the APTS Choice for Farmers of Different Scales

Table 5 shows that natural risks have significant differences in the selection of APTS for farmers of different scales (Model 6–Model 8), but have no effect on the type of APTS selection. Among them, natural risk has the greatest impact on the APTS selection behavior of medium-scale farmers, followed by small-scale farmers, and has no impact on large-scale farmers. This shows that among small and medium-sized farmers, the ability of medium-sized farmers to resist risks is the worst, while large-scale farmers may mainly derive their main income from agricultural production, and they have more agricultural machinery and more self-service. Natural risks have little impact on their choice of whole-process APTS.

The NAEI has no difference in the service selection of some links of farmers of different sizes. When considering whole-process APTS, the greater the NAEI of small-scale and medium-scale farmers is, the greater the probability of choosing whole-process APTS, and the impact of large-scale farmers is not obvious. This may be because large-scale farmers are mainly engaged in agricultural production and are less dependent on the NAEI.

#### 4.5.2. The Moderating Effect of the NAEI on the APTS Choice of Farmers of Different Scales

Table 6 Model 9~Model 11 Regression results show that the NAEI has a moderating effect on the choice of APTS for farmers of different sizes. The results show that the NAEI significantly affects the adjustment of APTS selection under natural risk for farmers of different sizes. Among the small-scale and medium-scale farmers, the interaction between natural risk and the NAEI has a positive impact on the choice of APTS, and the small-scale interaction is significant at the 5% level, the medium-scale interaction is significant at the 10% level, and the large-scale interaction is not significant. This shows that the larger the scale is, the smaller the positive adjustment effect of the NAEI on the negative impact of natural risks on farmers’ choice of APTS behavior, which indicates that the larger the scale is, the greater the loss in the face of natural risks. The NAEI can be adjusted to a certain extent. To address the risks of natural disasters to agricultural production, a variety of methods and approaches are needed.

## 5. Discussion

Through the research of this paper, it is found that non-farm employment income can effectively alleviate the negative impact of natural risks, which has certain practical significance for promoting the development of agricultural production hosting services. This study conclusion further validates the results of the paper by Cao Zhenglin et al. [81]. The scholar found that natural risks had a negative impact on farmers’ choice behavior, but it was not significant. This may be due to the limited amount of data and the region [82,83,84], but the sample size of this paper is only 200, and the region is selected from the Sichuan region of China, which is small and not a major grain producing area. Therefore, this paper makes the results more reliable through scientific regional selection and large sample data. At the same time, the existing studies only focus on the relationship between non-farm employment and agricultural production trusteeship ser-vices [71,85,86], but do not pay attention to the moderating effect of non-farm employment income. Through the research in this paper, it is found that non-farm employment income can effectively alleviate the negative impact of natural risks, which has certain practical significance for promoting non-farm employment of farmers and promoting the development of agricultural production hosting services.

The possible contributions of this paper are as follows. First, from the perspective of research, this paper explores the impact of natural risks on the decision-making of farmers’ APTS behavior from the perspective of risk. As the most difficult factor for farmers to control in agricultural production, natural risk is a factor that agricultural producers focus on when conducting agricultural production. In particular, the natural risks that occur in the process of farmers’ participation in agricultural productive services are borne by farmers themselves, which has an important impact on farmers’ decision-making on APTS. Second, this research highlights the regulatory role of non-agricultural employment in the decision-making of natural risks on farmers’ production trusteeship service behavior and provides a new solution to the negative impact of natural risks on farmers’ production trusteeship service decision-making. At the same time, the different decision-making behaviors of APTS modes, are systematically analyzed, the differences between natural risk and non-agricultural employment in the decision-making of farmers’ APTS behavior at different scales are revealed, and targeted countermeasures and suggestions are proposed. Third, in terms of research methods, considering the problems of endogeneity and data bias, the propensity score matching method is used to test the robustness of the results; therefore, the research in this paper is more scientific.

Despite its potential contribution, this study has several limitations. First of all, this paper uses cross-sectional data from the 2018 survey. Although the data content are very rich and have research value, if tracking data can be obtained to form continuous panel data, it will be more convincing to observe the dynamic changes in farmers’ APTS. In future research, panel data can be obtained for further verification through telephone return visits and tracking surveys of 2018 data. Secondly, in terms of variable settings, natural risks, such as drought, flood, and hail, are more common and are not distinguished in this paper. At the same time, the degree of disaster will be better if it can be distinguished. In follow-up studies, we can continue to improve the index quantification, decompose the natural risks, and focus on the degree of disaster of the sample; thirdly, the reduction in grain production is a trigger for disputes between farmers and APTS. A reduction of grain production is due to human or natural disasters will increase transaction costs. Non-agricultural employment is only one aspect that can promote farmers’ participation in APTS. The new variable agricultural insurance can be introduced to verify whether the addition of agricultural insurance will weaken the negative impact of natural risks on farmers’ choice of production trusteeship services, and provide new solutions to address the inhibitory impact of natural risks.

In the future, further research can be carried out from the following aspects: first, through telephone return visits and follow-up surveys of 2018 data, panel data can be obtained for further verification; Second, when continuing the research, improve the quantification of indicators, decompose natural risks, and focus on the extent of damage to the sample. Thirdly, new variable agricultural insurance can be introduced to verify whether the participation of agricultural insurance will weaken the negative impact of natural risks on farmers’ choice of production and custody services, and provide a new solution to solve the inhibiting impact of natural risks.

## 6. Conclusions

Based on the modified farmer utility model, this paper deduces the decision-making mechanism of farmers’ APTS from the dual mechanism of natural risk and NAEI and uses the survey data of 956 farmers in the three northeastern provinces to verify the objectivity of the conclusion by using Mlogit and PSM. The results show that: first, natural risks hinder farmers’ choice of APTS, but there is little difference in the choice of APTS type, that is, the partial link APTS or the whole-process APTS. Second, the NAEI has a positive impact on farmers’ choice of APTS, and plays an enhanced regulatory role in the impact of natural risks on the choice of APTS; finally, the robust impact is also the head of farmers characteristic variables and family endowment characteristics. Among them, the age, education level and relationship network of the head of farmers have a significant positive impact on the farmers’ APTS, and the family’s possession of agricultural machinery has a significant negative impact on the farmers’ choice of production trusteeship service. There are obvious differences in the choice of APTS type between farmers’ risk preferences and family production and operation ability. In addition, the robustness analysis revealed that there is scale heterogeneity in the impact of natural risks and the NAEI on the choice of APTS. The study revealed that natural risks and the NAEI have a significant impact on the choice of APTS for small-scale and medium-scale farmers. It has no effect on large-scale farmers.

According to the above research conclusions, some useful policy implications can be obtained in the promotion of the APTS: Firstly, transfer natural risks. Due to the particularity of agricultural production, farmers often face various uncertainties caused by natural risks in the production process. These risks may arise from a variety of factors such as climate change, pest and disease infestation, and changes in soil fertility, which not only affect the growth and yield of crops, but also directly affect the economic interests and quality of life of farmers. The APTS organizations can transfer risks by purchasing agricultural insurance, improving a reasonable insurance system, developing diversified agricultural insurance products, and establishing sound insurance claims supervision mechanisms.

Secondly, increasing non-agricultural employment opportunities. The government should actively stabilize the non-farm employment market for rural households, and create more non-agricultural employment opportunities so that rural households can have more choices and retreats when facing agricultural risks. At the same time, the government should also improve the ability of rural households to resist risks through various means, such as providing training on risk prevention and control, and promoting advanced agricultural technology, so that farmers can deal with risks more calmly.

Thirdly, in order to further improve the efficiency and quality of agricultural production, we should further improve the agricultural production trusteeship service industry. This includes strengthening the standardized management of service organizations, improving the professional quality of service personnel, and optimizing service processes.

Finally, market segmentation, and targeted promotion are important. Farmers of different sizes have different abilities to resist risks. APTS can introduce different service policies for farmers of different sizes, and mobilize the enthusiasm of farmers to participate in APTS. In addition, agricultural trusteeship service subjects can strengthen their relationships with farmers, build APTS relationships based on acquaintances, enhance the trust of farmers’ service subjects, and may also effectively resolve risks and promote farmers’ choice of APTS.

## Figures and Tables

**Table 1:** The meaning of each variable in the model and the results of descriptive statistical analysis.

Variable Category	Variable Name	Variable Definition	Mean	S.D.	Min	Max
Explained variables	APTS selection behavior (*Choose*)	Self-cultivation = 0; select partial link APTS = 1; choose whole-process APTS = 2	2.15	0.52	0	2
key variables	Natural risk (*Disaster*)	Was there a natural disaster last year: yes = 1, no = 0	0.54	0.50	0	1
	NAEI (*Nonfarm*)	Annual per capita NAEI of farmers: the sum of NAEI of each member last year divided by the total number of farmers population	6760.26	9922.78	0	87,500
control variable	Individual characteristic					
	Farmer age (*Age*)	The actual age of the head of farmer (years)	55.50	10.20	25	86
	Farmer education (*Education*)	years of education (years)	7.04	2.81	0	15
	Risk preference (*Risker*)	You are willing to give up your immediate interests for the long-term benefit: 0–10 points	4.48	3.00	0	10
	Family endowment variable					
	Net income (*Family income*)	The actual annual net income of the family (yuan)	59,951.07	61,677.19	1504	874,192
	Fluctuation of farmers production output (*Output*)	The difference between the highest yield and the lowest yield in the past 5 years (kg/mu)	511.21	282.99	34	2300
	Is there agricultural machinery (*Machinery*)	Agricultural machinery = 1, no agricultural machinery = 0	0.47	0.50	0	1
	Relational network (*Service*)	The relationship between the server and you: 0 = stranger, 1 = known person, 2 = relative.	0.82	0.50	0	2
	Area dummy variables					
	Province variables (*Province*)	Heilongjiang = 1, Jilin = 2, Liaoning = 3	1.88	0.76	1	3
	Region variable (*City*)	Harbin = 1, Suihua = 2, Qiqihar = 3, Changchun = 4, Siping = 5, Tieling = 6	4.05	1.55	1	6

**Table 2:** Estimated results of the impact of natural risks and the NAEI on APTS choice behavior.

Variable Name	Partial Link APTS			Whole-Process APTS		
	Model 1	Mode 2	Mode 3	Mode 1	Mode 2	Mode 3
*Disaster*	−0.8478 ** (0.3473)	—	−0.7672 ** (0.3490)	−0.9415 ** (0.3724)	—	−0.8628 ** (0.3738)
*Nonfarm*	—	0.4798 ** (0.2270)	0.4213 * (0.2288)	—	0.4956 ** (0.2386)	0.4358 * (0.2401)
*Age*	0.3965 ** (0.1729)	0.4220 ** (0.1741)	0.4145 ** (0.1740)	0.4470 ** (0.1860)	0.4708 ** (0.1869)	0.4651 ** (0.1868)
*Education*	0.2995 * (0.1689)	0.3070 * (0.1693)	0.2942 * (0.1703)	0.3631 ** (0.1814)	0.3665 ** (0.1820)	0.3540 * (0.1827)
*Risker*	0.0828 (0.1648)	0.0973 (0.1640)	0.1040 (0.1658)	0.3559 ** (0.1771)	0.3755 ** (0.1764)	0.3791 ** (0.1780)
*Family* *income*	−0.0383 (0.1770)	−0.1626 (0.1728)	0.1587 (0.1639)	0.0409 (0.1861)	−0.0903 (0.1803)	−0.0825 (0.1707)
*Output*	0.3086 * (0.1720)	0.2254 (0.1646)	0.2788 (0.1728)	0.3648 ** (0.1814)	0.2766 (0.1748)	0.3321 * (0.1825)
*Machinery*	−0.7693 ** (0.3843)	−0.7635 ** (0.3816)	−0.6907 * (0.3886)	−1.6846 *** (0.4116)	−1.6873 *** (0.4091)	−1.6060 *** (0.4154)
*Service*	4.6673 *** (0.5421)	4.6014 *** (0.5402)	4.6495 *** (0.5439)	4.1623 *** (0.5608)	4.0996 *** (0.5596)	4.1413 *** (0.5630)
Area dummy variables	controlled	controlled	controlled	controlled	controlled	controlled
*Constant*	3.3526 *** (0.7248)	2.5413 *** (0.6416)	3.2066 *** (0.7260)	1.3394 * (0.7736)	0.4616 (0.6919)	1.1964 (0.7747)
*Log likelihood*	−521.6670	−522.6065	−519.7582	−521.6670	−522.6065	−519.7582
*Prob* > *chi*2	0.0000	0.0000	0.0000	0.0000	0.0000	0.0000

Note: *, **, *** are significant at the levels of 10%, 5% and 1% respectively, and the number in brackets is the standard error of the coefficient.

**Table 3:** Estimated results of the impact of APTS on farmers’ choice of APTS.

Variable Name	Model 5: Partial Link APTS		Model 5: Whole-Process APTS	
	Coefficient	Standard Error	Coefficient	Standard Error
*Disaster*	−0.6960 *	0.3596	−0.7896 **	0.3834
*Nonfarm* × *Disaster*	0.6396 **	0.3249	0.5978 *	0.3399
*Age*	0.4186 **	0.1746	0.4688 **	0.1873
*Education*	0.3053 *	0.1710	0.3704 *	0.1831
*Risker*	0.0726	0.1663	0.3464 *	0.1783
*Family income*	−0.1441	0.1639	−0.0503	0.1694
*Output*	0.2569	0.1719	0.3142 *	0.1813
*Machinery*	−0.7509 *	0.3894	−1.6728 ***	0.4158
*Service*	4.6616 ***	0.5443	4.1555 ***	0.5633
Area dummy variables	controlled	controlled	controlled	controlled
*Log likelihood*	−519.46306			
*Prob > chi*2	0.0000			
Adjust *R*^2^	0.2926			
*N*	949			

Note: *, **, *** are significant at the level of 10%, 5% and 1%, respectively.

**Table 4:** Considers the robustness test of self-selection bias (PSM).

Variable Name	Matching Method		Treatment Group	Control Group	ATT	S.D.	T Value
Natural risk	K neighborhood matching (k = 4)	Before matching	0.909	0.949	−0.041	0.017	−2.40 **
		After matching	0.911	0.959	−0.0482	0.020	−2.44 **
	Radius matching (0.01)	Before matching	0.909	0.949	−0.041	0.017	−2.40 **
		After matching	0.911	0.951	−0.040	0.019	−2.12 **
	Kernel matching	Before matching	0.909	0.949	−0.041	0.017	−2.40 **
		After matching	0.911	0.948	−0.038	0.018	−2.05 **
Non-agricultural employment	K neighborhood matching (k = 4)	Before matching	0.945	0.903	0.041	0.017	2.44 **
		After matching	0.945	0.878	0.067	0.025	2.72 ***
	Radius matching (0.01)	Before matching	0.945	0.903	0.041	0.017	2.44 **
		After matching	0.944	0.893	0.051	0.024	2.11 **
	Kernel matching	Before matching	0.945	0.903	0.041	0.017	2.44 **
		After matching	0.945	0.885	0.059	0.022	2.66 **

Note: **, *** are significant at the level of 5% and 1% respectively, and the number in brackets is the standard error of the coefficient.

**Table 5:** Estimation results of the impact of natural risks and the NAEI on the choice of the APTS for different sizes of farmers.

Variable Name	Partial Link APTS			Whole-Process APTS		
	Model 6	Mode 7	Mode 8	Mode 6	Mode 7	Mode 8
	Small-Scale Farmers	Medium-Scale Farmers	Large-Scale Farmers	Small-Scale Farmers	Medium-Scale Farmers	Large-Scale Farmers
Natural risk	−1.6427 * (0.8656)	−1.7935 ** (0.7561)	−0.7339 (0.7651)	−1.5571 * (0.8706)	−2.2812 *** (0.7908)	−0.0604 (1.0118)
NAEI	1.3884 ** (0.6381)	1.1737 * (0.6452)	0.7927 ** (0.4045)	1.5067 ** (0.6506)	1.3407 ** (0.6617)	0.6469 (0.4857)
Control variables	controlled	controlled	controlled	controlled	controlled	controlled
*Log likelihood*	−208.2572	−190.0261	−80.84248	−208.2572	−190.0261	−80.84248
*Prob* > *chi*2	0.0000	0.0000	0.0000	0.0000	0.0000	0.0000
Adjust *R*^2^	0.2914	0.2858	0.5006	0.2914	0.2858	0.5006
*N*	382	363	211	382	363	211

Note: *, **, *** are significant at the levels of 10%, 5% and 1%, respectively, and the numbers in parentheses are the standard errors of the coefficients. The control variables include the characteristic variables, family endowment variable and Area dummy variables.

**Table 6:** Estimated results of the impact of non-agricultural employment adjustment on the choice of the APTS for different sizes of farmers.

Variable Name	Partial Link APTS			Whole-Process APTS		
	Model 9	Model 10	Model 11	Model 9	Model 10	Model 11
	Small-Scale Farmers	Medium-Scale Farmers	Large-Scale Farmers	Small-Scale Farmers	Medium-Scale Farmers	Large-Scale Farmers
Natural risk	−1.7134 ** (0.8682)	−1.5279 ** (0.7719)	−1.0751 (0.7504)	−1.6192 ** (0.8606)	−2.0277 ** (0.8060)	−0.3296 (1.0069)
NAEI	1.0685 ** (0.2272)	1.2155 * (0.7362)	0.4430 (0.4821)	1.1278 ** (0.3516)	1.2578 * (0.7535)	0.7695 (0.6443)
Control variables	controlled	controlled	controlled	controlled	controlled	controlled
*Log likelihood*	−210.2235	−190.9400	−82.375615	−210.2235	−190.9400	−82.375615
*Prob* > *chi*2	0.0000	0.0000	0.0000	0.0000	0.0000	0.0000
Adjust *R*^2^	0.2847	0.2823	0.4912	0.2847	0.2823	0.4912
*N*	382	363	211	382	363	211

Note: *, **, are significant at the levels of 10%, 5% respectively, and the number in brackets is the standard error of the coefficient. The control variables include the characteristic variables, family endowment variable and Area dummy variables.

## Data Availability

The original contributions presented in the study are included in the article, further inquiries can be directed to the corresponding author.
